# Biomonitoring of Toxic Metals in Feathers of Birds from North-Eastern Pakistan

**DOI:** 10.1007/s00128-021-03184-w

**Published:** 2021-03-20

**Authors:** Bushra Aziz, Muhammad Zubair, Nausheen Irshad, Khawaja Shafique Ahmad, Majid Mahmood, Majid Mahmood Tahir, Khizar Hussain Shah, Aqeela Shaheen

**Affiliations:** 1 Department of Zoology, University of Poonch Rawalakot (UPR), Rawalakot, 12350 Azad Jammu and Kashmir Pakistan; 2 Department of Veterinary Clinical Sciences, University of Poonch Rawalakot, Rawalakot, Azad Jammu and Kashmir Pakistan; 3 Department of Botany, University of Poonch Rawalakot (UPR), Rawalakot, 12350 Azad Jammu and Kashmir Pakistan; 4 Department of Soil and Environmental Science, University of Poonch Rawalakot (UPR), Rawalakot, 12350 Azad Jammu and Kashmir Pakistan; 5 Department of Chemistry, Comsats University Islamabad, Abbottabad Campus, Abbottabad, KPK Pakistan

**Keywords:** Heavy metals, Birds, Feathers, Poonch, Azad Kashmir

## Abstract

The current study was designed to determine the concentrations of toxic metals (Ni, Pb and Cr) in feathers of birds collected from four regions of NE Pakistan. Feather samples of birds (House Crow, Common Myna and House Sparrow) were collected from different areas. Atomic absorption spectrophotometer was used to determine the concentration of metals in feathers. Analysis of the data revealed that concentrations of Pb and Cr were significantly different (*p* < 0.05) among bird species, whereas no difference (*p* > 0.05) was detected among bird species (house crow, common myna and house sparrow) for Ni. A significant difference was found for the concentration of Pb and Ni in all the four studied regions. Whereas, non-significant difference was found in all the studied regions for the concentrating of Cr. It was revealed that there is significant rising concentration of metals (Pb, Cr) in feathers of birds in Azad Kashmir.

Heavy metals exist in nature in minute quantities, but industrialization and certain urbanization practices have increased their levels in the environment posing to threat to wild life (Pollack et al. 2017). Important anthropogenic origins of heavy metals include city progress and various related activities such as road traffic, biomass and solidified goods for combustion (Agarwal 2002). These heavy metals include mercury (Hg), cadmium (Cd), chromium (Cr), lead (Pb), nickel (Ni), cobalt (Co), and zinc (Zn). However, at high levels they can be toxic for organisms. (Gerbersmann et al. 1997; Lee et al. 2006). A considerable amount of heavy metals is released into the atmosphere by city traffic and it can get deposited into the roadside soil, which finally becomes a source of heavy metal exposure (Manta et al. 2002; Sternbeck et al. 2002; Mohanraj et al. 2004). Furthermore, human activities produce large amount of metals that are released directly into the environment, polluting ecosystems, food webs etc. beyond human limits (Alloway 1990; Dudka and Miller 1999; Metchevas et al. 2010).

Metals not only reduce energy levels in humans, but also impair the functions of many of the body's essential organs, including kidney, brain, heart and lungs. Long-term heavy metal exposure causes muscle degeneration, and physical and neurological disorder, leading to Parkinson's disease (Scorza et al. 2017).

Wildlife species are adversely affected by heavy metals (Scheuhammer 1987; Janssens et al. 2003). The adverse effects of these metals on wildlife worldwide are a growing concern because of industrialization. Harmful chemicals and heavy metals that are released into the atmosphere from factories and other human activities contaminate our climate, food and water resources (Hamidullah et al. 1997; Yousafzai and Shakoori 2008).

The accumulation of heavy metals depends on the physiology of the species, the metals' properties and their availability/accessibility in the environment. After ingestion, heavy metals accumulate in different animal body tissues, such as the feathers of birds (Furness et al. 1986). Feathers are associated with the blood through the blood vessels (Dauwe et al. 2003), and are a good indicator of heavy metals deposited in the blood as such (Dauwe et al. 2003; Burger 1993). In order to control the environmental concentration of heavy metals, it is important to select birds that are sensitive to changes in the environment and those that respond in predictable ways.

For this purpose, House Sparrow *Passer domesticus*, House Crow *Corvus splendens* and Common Myna *Acridotheres tristis* were used in the current study to monitor metal contamination. These birds are related and usually live within human inhabitation. Such birds also feed on waste from human habitats, small animals, fruit, etc. (Hunt 2016).

No information on environmental pollution in urban hilly areas of Pakistan is available to date. The current study aimed to quantify the heavy metals (lead, chromium and nickel) concentrations in the feathers of House Crow, Myna and Sparrow from District Poonch, to assess feasibility as a biomonitoring agent in assessing environmental hazards of heavy metals pollution in urban areas. The second objective of the current study was to compare the results of this study with the recorded values in Pakistan's polluted area, as well as in some other countries in order to learn more about the extent of the impact of metal pollution.

## Materials and Methods

**Fig. 1:**
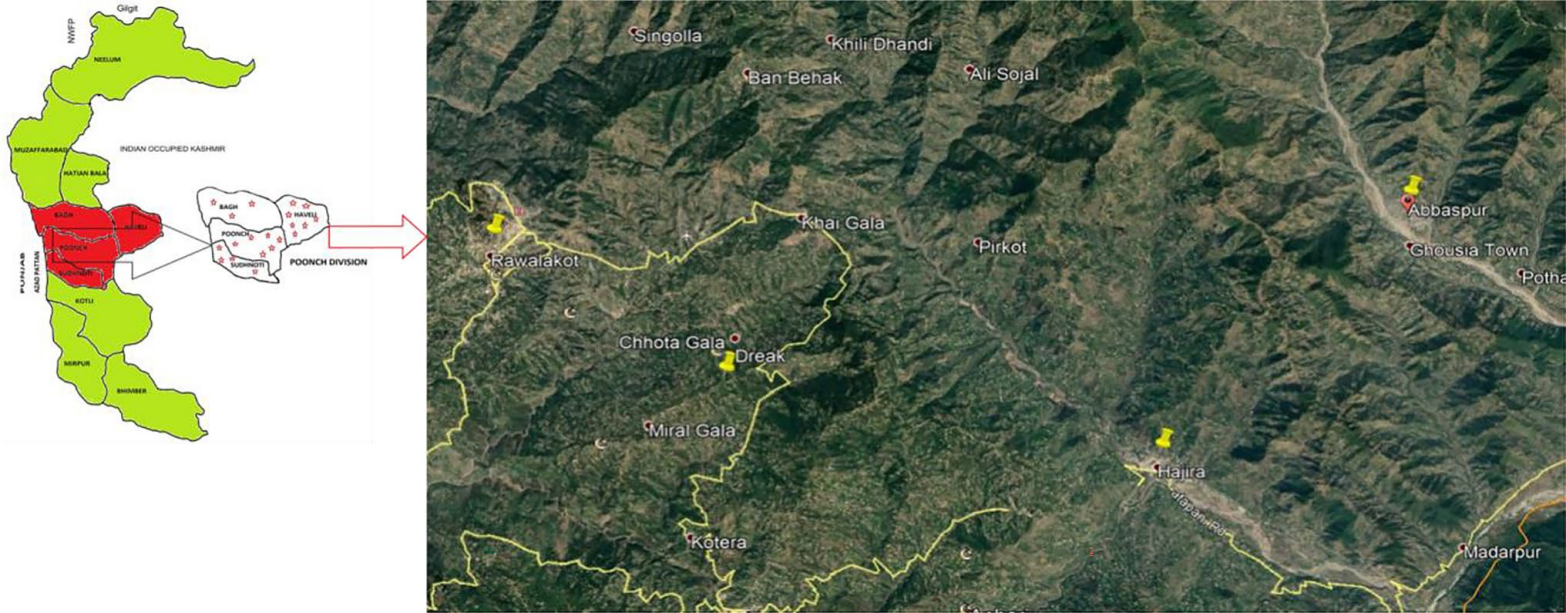
Figure indicating the sample sites

Azad Kashmir has ten districts and Poonch is one of these districts. District Poonch has four tehsils Rawalakot, Thorar, Hajira and Abbaspur. It is situated among longitude of 33.85°N and latitude of 73.75°E. No data about the use environmental pollutants like insecticides, fertilizers and chemicals is available in this area. Feathers were collected from four regions (Rawalakot, Hajira, Abbaspur and Dreak) of district Poonch Azad Jammu and Kashmir during the study period (March 2018–Nov 2018) by using nets at their roosting sites. Sample sites are presented in Fig. 1. Three to four breast and tail plumage were collected from each bird species and were placed in a sealed plastic bag.

Feathers obtained from collected samples were first wiped three times with tap water, splashed with distilled water, and then cleaned in acetone (Saeki et al. 2000; Battaglia et al. 2003), to ensure there was no external pollutant present (Goede and Bruin 1984). Thereafter, samples were dried in an oven for 8 h at 80°C (Saeki et al. 2000). Feathers were dissected into small pieces and samples were weighed in grams and transferred into quartz crucibles. In the quartz crucibles, 2 mL of HNO_3_ and percholric acid 0.5 mL added and covered with watch glasses. A magnetic hot plate was used for digestion at both temperatures (initially at low temperature and then at elevated temperature). The dilution of digested samples was carried out by adding 10 mL de-ionized water. To determine the concentration of heavy metals, digested samples were analyzed in triplicate using an atomic absorption spectrophotometer. The analysis of samples was carried out by using the flame atomic absorption spectrophotometer (Perkin-Elmer-AAS-800). Aliquots of sample was prepared to make the quality control (QC) sample, this was representative of whole sets of sample. This QC sample was injected after every 15 min in order to assess the stability of this instrument. There were less than 15% variations of metals concentration in the QCs. A reagent blank was also used in order to find the containments in different extracts. Relative standard deviation ranged from 5% to 10% in this experiment and calculated from standard deviation by dividing the mean. Different concentrations of standard solutions i.e. 0.5 ppm, 1 ppm, 3 ppm and 5 ppm were used to for calibration curve in this experiment. The concentration of this experiment was set to zero using the reagent blank. All the reagent and stock solutions used in this experiment was obtained from Merck having the analytical grade.

Statistical analysis was carried by using the software SPSS (version 16.0). The concentrations of metal ppm were not normally distributed (Shapiro–Wilk test, *p* < 0.05); as a result, nonparametric statistic we used in this data. Kruskal–Wallis one-way analysis of variance was applied to find the differences in metal concentrations in regions and birds. On difference values, comparisons were made by Mann–Whitney U test. Pearson's correlation test was used to determine the correlation between different combinations of metals. Statistical package for social sciences (SPSS16.0) was used for all analyses.

## Results

A total of 108 samples were taken from three bird species including Crow, Myna and Sparrow. Out of these, 27 feather samples of House Crow from four regions were analyzed for metals concentration. Concentrations of metals and sample size are presented in Table 1 from different regions.

**Table 1:** Mean concentrations (± SD) ppm of metals (Pb, Ni, Cr) estimated from the feathers samples of three bird species (House Crow, Common Myna and House Sparrow)

Sr. no	Area	Name of bird	Sample size	Metal concentrations (mg/L)		
				Lead	Nickel	Chromium
1	Rawalakot	House Crow	9	0.740 ± 0.48	0.182 ± 1.05	0.207 ± 0.03
2	Rawalakot	Myna	9	1.761 ± 0.71	0.897 ± 0.62	0.225 ± 0.02
3	Rawalakot	House Sparrow	9	0.545 ± 0.50	0.611 ± 0.53	0.165 ± 0.05
4	Hajira	House Crow	9	0.785 ± 0.58	0.760 ± 0.84	0.178 ± 0.05
5	Hajira	Myna	9	0.643 ± 0.17	0.322 ± 0.17	0.251 ± 0.02
6	Hajira	House Sparrow	9	2.253 ± 1.57	1.362 ± 1.39	0.212 ± 0.32
7	Abbaspur	House Crow	9	0.024 ± 0.11	0.276 ± 0.69	0.159 ± 0.04
8	Abbaspur	Myna	9	0.191 ± 0.70	0.104 ± 0.60	0.260 ± 0.16
9	Abbaspur	House Sparrow	9	1.157 ± 0.67	0.039 ± 0.46	0.181 ± 0.02
10	Dreak (Rawalakot)	House Crow	9	1.048 ± 0.89	1.700 ± 0.76	0.256 ± 0.14
11	Dreak (Rawalakot)	Myna	9	1.064 ± 0.45	0.848 ± 0.56	0.261 ± 0.09
12	Dreak (Rawalakot)	House Sparrow	9	0.729 ± 0.47	0.052 ± 0.41	0.202 ± 0.04

Collected during the current study from different regions of District Poonch Azad Kashmir

*NA* not analyzed

Metals concentration after analysis is presented in Table 2. Ni (mean ± SD 1.70 ± 0.76 ppm) was highly present in samples collected in the Dreak region, followed by Pb (1.04 ± 0.8 ppm) and Cr (0.25 ± 0.13 ppm) (Table 2). For common myna, Pb was high in samples collected from the Rawalakot region (76 ± 0.7 ppm). Ni (0.89 ± 0.62 ppm) was also highly deposited in collected feathers from the Rawalakot region. The lowest concentration of Cr (0.26 ± 0.08 ppm) was observed in the feathers collected from the Myna region and those collected from the Dreak region. Feather samples of House Sparrow from four studied regions were analyzed to estimate the concentration of heavy metals. In all the studied metals Pb (2.25 ± 1.57 ppm) was highly deposited in House Sparrows of the Hajira region (Table 1) followed by Ni (1.36 ± 1.39 ppm) and Cr (0.21 ± 0.03 ppm).

**Table 2:** Mean concentrations (± SE) ppm of heavy metals concentration in House Crow, Common Myna and House Sparrow among the study sites

	Rawalakot	Hajira	Abbaspur	Dreak	Kruskal–Wallis test on difference (*p* value)
House Crow					
Lead (ppm)	0.74 ± 0.48^AB^	0.78 ± 0.58^A^	0.02 ± 0.10^B^	1.05 ± 0.89^A^	**0.006**
Nickel (ppm)	0.18 ± 1.05^A^	0.76 ± 0.84^AB^	0.27 ± 0.69^A^	1.70 ± 0.77^B^	**0.000**
Chromium (ppm)	0.21 ± 0.03	0.18 ± 0.05	0.16 ± 0.04	0.26 ± 0.14	0.070
Myna					
Lead (ppm)	1.76 ± 0.71^A^	0.64 ± 0.17^BC^	0.19 ± 0.70^C^	1.06 ± 0.45^AB^	**0.000**
Nickel (ppm)	0.90 ± 0.62^A^	0.05 ± 0.16^B^	0.08 ± 0.60^B^	0.85 ± 0.56^A^	**0.000**
Chromium (ppm)	0.22 ± 0.01	0.25 ± 0.02	0.26 ± 0.16	0.26 ± 0.08	0.837
House Sparrow					
Lead (ppm)	0.54 ± 0.5^A^	2.25 ± 1.57^B^	1.15 ± 0.67^AB^	0.72 ± 0.47^A^	**0.002**
Nickel (ppm)	0.61 ± 0.52^AB^	1.36 ± 1.39^B^	0.04 ± 0.47^A^	0.05 ± 0.40^A^	**0.004**
Chromium (ppm)	0.16 ± 0.05	0.21 ± 0.03	0.18 ± 0.02	0.20 ± 0.03	0.057
Overall					
Lead (ppm)	1.01 ± 0.78^AB^	1.23 ± 1.19^A^	0.46 ± 0.74^B^	0.95 ± 0.64^AB^	**0.012**
Nickel (ppm)	0.56 ± 0.80^A^	0.60 ± 1.15^A^	0.05 ± 0.59^B^	0.87 ± 0.89^A^	**0.001**
Chromium (ppm)	0.20 ± 0.04	0.21 ± 0.04	0.20 ± 0.10	0.24 ± 0.10	0.198

Similar superscripts in row are considered as non significant

Kruskal–Wallis test and Mann–Whitney U test was applied to find the differences in metal concentrations in regions and birds species (House Crow, Common Myna and House Sparrow) for Pb, Cr and Ni (Table 3). Concentrations of Pb and Cr were significantly different (*p* < 0.05) among bird species (House Crow, Common Myna and House Sparrow). There were no differences detected among bird species (House Crow, Common Myna and House Sparrow) for Ni. The same superscript in each row indicates that the groups do not differ.

**Table 3:** Species difference in concentration (± SE) of metals

Metals	House Crow	Myna	House Sparrow	Kruskal–Wallis test on difference (*p* value)
Lead	0.65 ± 0.68^B^	0.91 ± 0.79^AB^	1.17 ± 1.11^A^	**0.046**
Nickel	0.59 ± 1.10	0.33 ± 0.75	0.52 ± 0.95	0.483
Chromium	0.20 ± 0.08^B^	0.25 ± 0.09^A^	0.19 ± 0.04^B^	**0.002**

Different letters indicate significant differences in rows

The concentration of Pb, Cr and Ni differed among bird species (House Crow, Common Myna and House Sparrow) from Rawalakot, Hajira, Abbaspur and Dreak region (Table 3). A significant difference (*p* < 0.05) was found for the concentration of Pb and Ni in all the four studied regions. No difference was detected for the concentrating of Cr among regions.

Concentrations of trace metals were significantly correlated in some cases (Table 4). In House Crow for example, Pb was significantly correlated with Ni and Cr. Similarly, there was significant correlation between Ni and Cr. Correlation coefficient was 0.598 and 0.214 and 0.273, respectively. In the feather samples of Common Myna, Pb was significantly correlated with Ni. However, there was negative correlation between Pb and Cr as well as between Ni and Cr. However, there was a significant correlation between all three metals in House Sparrow.

**Table 4:** Pearson's correlation coefficients between different combinations of metals in all three species of birds

Species	Metal combinations	Correlation coefficient	*p*-value
Overall	Pb–Ni	0.598	**0.000**
	Pb–Cr	0.214	**0.026**
	Ni–Cr	0.273	**0.008**
Crow	Pb–Ni	0.617	**0.000**
	Pb–Cr	0.469	**0.004**
	Ni–Cr	0.642	**0.000**
Myna	Pb–Ni	0.586	**0.000**
	Pb–Cr	− 0.060	0.728
	Ni–Cr	− 0.019	0.912
Sparrow	Pb–Ni	0.720	**0.000**
	Pb–Cr	0.589	**0.000**
	Ni–Cr	0.313	0.063

## Discussion

Environmental pollution is one of the most significant ecological crises the world facing today (Karan and Harada 2001). Metals for instance, can be introduced into the environment from anthropogenic sources and are harmful to most of the organisms in excess (Guven et al. 1999). It is therefore important to quantify heavy metals concentrations in the environment in a wide variety of wildlife. For this purpose, wild birds are used as a bioindicator of metal exposure in the natural environment. Wild birds living in close association with human habitation have the highest risk of being affected by the pollutants and are a useful bioindicator of environmental pollution. House Crow, Common Myna and House Sparrow are ideal for these types of monitoring programs.

Metals are vital for different biological functions such as zinc (i.e. essential metals), but high levels can be lethal for organisms. Pb is a heavy metal which is dangerous for wildlife and human health, and this metal can be extremely harmful (Roux and Marra 2007). Food and water intake of Pb may be fatal to birds (Scheuhammer 1987). It interferes with their endocrine system (Stocia et al. 2000; Martin et al. 2003), kidney activity (Nordberg 1971), reproduction (Buerger et al. 1986; Hui 2002).

More consumption of Pb from food and water can be deadly to the birds (Scheuhammer 1987). The threshold level of Pb is 2 ppm in the liver of birds (Pain et al. 1995). The mean concentration of Pb varies from 0.024 ± 0.10 to 2.25 ± 1.57 ppm in the present study, which is much lower than that needed to elicit adverse effects. Analyzing Pb from different feathers in Calamus and Vane, Yamac et al. (2019) reported the value of 0.65 ppm and 5.47 ppm, respectively. Concentration of Pb reported from other regions of Pakistan (Abdullah et al. 2015; Malik and Zeb 2009; Ullah et al. 2014; Nighat et al. 2013) were lower than findings here. The reason for the lower concentration is likely due to lower industrial activities in these areas. In another study, reported concentration of Pb ranged from 1.15 to 2.3 ppm (Gruz et al. 2019). There was a significant rise of Pb in the feathers of Sparrow in Hajira as compared to other two birds in present study. The rise of Pb in Hajira in our study can also be attributed to rise of traffic activities in the city area. It has been reported that major sources of Pb poising are due to contaminated soil, smelters and contaminated carcass (Legagneux et al. 2014; Gruz et al. 2019).

Ni enters into the brain, liver, kidney, bones, heart and endocrine glands. It can also be deposited into the nails, hair and saliva (Duda-Chodak and Blaszczyk 2008). The ingestion of Ni by birds and wildlife affects their respiratory system and results in asthma and also destroys the DNA. Concentration of Ni varies from 0.027 ± 0.69 to 1.70 ± 0.79 ppm in the present study in three birds. There was a similar study conducted by Manpreet and Khera (2018) on the Crow. The authors measured Ni from two districts (Ludhiana and Sangrur) of Punjab, India. According to their findings, the concentration of Ni ranged from 0.625–4.2 ppm to 8.45–13.8 ppm in studied areas. There was significant rise of Ni in Dreak regions as compared to other regions. Whereas, the lowest concentration of Ni was (12.29 ppm) in all the observed areas.

In some other studies of Pakistan, 30–47.5 ppm and 77–89 ppm concentration of Ni were reported in birds from the Lahore and Sialkot districts (Abdullah et al. 2015). This concentration is higher than our study. Similar values of Ni were reported in some other areas of Pakistan (Boncompagni et al. 2003; Malik and Zeb 2009; Ullah et al. 2014; Nighat et al. 2013). It was suggested that industrial activities including Ni–Cr platting, ghee production and electrical manufacturing are considered as the major rise of this metal (Hanif et al. 2005). Reduced levels reported here for metals may be due to absence of these activities as the area investigated here is high altitude. According to many studies, heavy metals adversely influence wildlife species (Scheuhammer 1987; Janssens et al. 2003) and this has become a worldwide problem because of industrialization. Our air, food and water are contaminated by harmful chemicals and heavy metals that are released into the environment by industries and other human activities (Hamidullah et al. 1997; Yousafzai and Shakoori 2008).

Chromium is considered as the non essential element of animals with major disturbances in reproductive health of avian species (Malik and Zeb 2009). There are few reports available worldwide on level of chromium in avian species. There was a similar study conducted on the Crow (Manpreet and Khera 2018). They observed the presence of Cr in birds from two districts (Ludhiana and Sangrur) of Punjab, India. As well, the concentration of Cr ranged from 1.1–4.9 ppm in Ludhiana district and 7.45–14.15 ppm in Sangrur district. The mean concentrations of Cr of present study were lower than those found in feathers of Crow of Punjab, India.

Similarly, some high values of Cr were reported in avian feathers in Pakistan and China (Burger and Gochfeld 1995; Ullah et al. 2014; Deng et al. 2007; Zhang et al. 2006). According to the current study, Hajira was found to be more polluted than other studied areas. Higher concentration of this metal is also reported in feathers of little egrets from Taunsa, Pakistan (Boncompagni et al. 2003). These study areas are famous for the leather industry and higher concentration may be due to this industry. The low concentration of Cr in present study may be due to absence of this industry. High concentrations of Cr in the region of Hajira may be due to some migration of birds from the contaminated area. Moreover, urbanization also leads to accumulate the higher level of metals in birds (Bichet et al. 2013; Meillère et al. 2016).

Significant correlations were recorded in House Crow, Common Myna and House Sparrow for Ni and Pb concentrations and in House Crow and House Sparrow between Pb and Cr in relation to the correlations of toxic metals within species in the current study. Correlation in the concentration of metals have been reported for Cd and Pb concentration in feathers by Zarrintab et al. (2016), in the calamus by Yamac et al. (2019) and between As and Cd concentration in the Vane (Yamac et al. 2019).

Similarly, Cd was significantly correlated with As and Pb in owls with values of 0.43 ppm and 0.38 ppm, respectively (Grúz et al. 2019). These correlations of present study indicate that these metals are interlinked with each other during contamination.

The current study concludes that bird feather testing is a valuable tool for tracking the occurrence of trace metals found in the environment and the accumulation of metals in birds in regions of Pakistan. More research is required, however, to explore whether feathers can be useful for monitoring the internal concentrations of other contaminant groups. Yet, there are still many knowledge gaps that need to be systematically and experimentally addressed in order to conclude that feathers are suitable as a conduit for other forms of contaminants.
